# Clinical impact of high-attenuation and cystic areas on computed tomography in fibrotic idiopathic interstitial pneumonias

**DOI:** 10.1186/s12890-015-0069-0

**Published:** 2015-07-24

**Authors:** Kiminobu Tanizawa, Tomohiro Handa, Sonoko Nagai, Toyohiro Hirai, Takeshi Kubo, Tsuyoshi Oguma, Isao Ito, Yutaka Ito, Kizuku Watanabe, Kensaku Aihara, Kohei Ikezoe, Toru Oga, Kazuo Chin, Takateru Izumi, Michiaki Mishima

**Affiliations:** Department of Respiratory Care and Sleep Medicine, Graduate School of Medicine, Kyoto University, Kyoto, Japan; Department of Respiratory Medicine, Graduate School of Medicine, Kyoto University, 54 Shogoin Kawaharacho, Sakyo-ku, Kyoto, 606-8507 Japan; Kyoto Central Clinic, Clinical Research Center, Kyoto, Japan; Department of Diagnostic Imaging and Nuclear Medicine, Graduate School of Medicine, Kyoto University, Kyoto, Japan; Department of Respiratory Medicine, Allergy and Clinical Immunology, School of Medical Sciences, Nagoya City University, Nagoya, Japan; Department of Respiratory Medicine, Fukui Red-Cross Hospital, Fukui, Japan; Department of Respiratory Medicine, Saiseikai-Noe Hospital, Osaka, Japan

**Keywords:** Quantitative computed tomography, Densitometry, Idiopathic interstitial pneumonias, Prognosis

## Abstract

**Background:**

Quantitative computed tomography (CT) analysis has been proposed as a means of objectively assessing fibrotic interstitial pneumonia (IP) including idiopathic pulmonary fibrosis (IPF). We investigated whether percentages of high-attenuation areas (HAA%) and cystic areas (CA%) quantified from CT images were useful as indices of fibrotic IP.

**Methods:**

CT images of 74 patients with fibrotic idiopathic interstitial pneumonias (IPF, 36; non-specific interstitial pneumonia, 9; unclassifiable idiopathic interstitial pneumonia, 29) were analyzed via in-house computer software, which automatically calculated HAA%, CA%, mean lung density (MLD), standard deviation of lung density (SD-LD), kurtosis, and skewness from CT attenuation histograms. These indices were compared in each instance with physiologic measures, visual fibrosis score, clinical diagnosis, radiologic CT pattern, and prognosis.

**Results:**

HAA% correlated significantly with physiologic measures and visual fibrosis score to a moderate extent (%forced vital capacity, r_s_ = −0.59; % carbon monoxide diffusion capacity, r_s_ = −0.43; fibrosis score, r_s_ = 0.23). Densitometric parameters (MLD, SD-LD, kurtosis, and skewness) correlated significantly with physiologic measures and fibrosis score (|r_s_| = 0.28-0.59). CA% showed no association with pulmonary functions but differed significantly between IPF and other interstitial pneumonias (IPs) (1.50 ± 2.41 % vs. 0.41 ± 0.80 %; *P* < 0.01) and between the definite usual interstitial pneumonia (UIP) pattern and other patterns (1.48 ± 2.38 % vs. 0.55 ± 1.19 %; *P* < 0.01). On univariate analysis, HAA%, MLD, SD-LD, kurtosis, skewness, fibrosis score, and definite UIP pattern all correlated with survival, with kurtosis alone identified as a significant predictor of mortality on multivariate analysis (hazard ratio = 0.67; 95 % CI, 0.44-0.96; *P* = 0.03).

**Conclusion:**

CA% and HAA% are novel quantitative CT indices with differing properties in fibrotic IP evaluations. HAA% largely reflects physiologic impairments, whereas CA% corresponds with diagnosis and HRCT pattern. Of the CT indices examined, kurtosis constituted the strongest predictor of mortality.

## Background

Quantitative high-resolution computed tomography (HRCT) analysis of the lung has been proposed as an objective and non-invasive means of assessing parenchymal lesions in fibrotic interstitial lung diseases (ILDs) [1–5]. Densitometric parameters (such as mean lung density [MLD], standard deviation of lung density [SD-LD], kurtosis, and skewness) derived from CT attenuation histograms have served as indices in earlier studies [1–6], corresponding with histopathologic diagnosis [3], physiologic impairment [1, 2, 4, 6], and health-related quality of life [4] in fibrotic ILDs, and with survival in idiopathic pulmonary fibrosis (IPF) [5]. However, no standard quantitative method for assessing fibrotic ILDs (IPF and others) by CT has been adopted as yet, nor has the clinical utility of this approach been firmly established [7].

Fibrotic ILDs are marked by a combination of radiologic abnormalities, with high and low CT attenuation [1]. Typically, high-attenuation areas (HAAs) signify parenchymal lesions, such as ground-glass opacity (GGO) and reticulation [8], whereas emphysematous change and cystic areas (CAs) are signified by low-attenuation areas (LAAs). CAs with honeycombing are more characteristic of fibrotic ILDs [8]. Determining percentages of HAAs (HAA%) and CAs (CA%) in whole lung fields may aid in determining the extents these characteristic lesions. It was our view that HAA% and CA% might serve as quantitative CT indices of fibrotic ILDs.

The main purpose of the present study was to determine the clinical utility of HAA% and CA% (alongside densitometric parameters) in assessing fibrotic interstitial pneumonia (IP). The relationships of these CT indices with physiologic impairment, visual score, clinical diagnosis, CT pattern, and prognosis were examined.

## Methods

### Patients

For this retrospective study, 74 consecutive patients with fibrotic idiopathic interstitial pneumonias (fibrotic IIPs) were recruited. All patients were undergoing HRCT at Kyoto Central Clinic, Kyoto, Japan between January 2004 and December 2006 and were followed for >3 months. Fibrotic IIPs comprised IPF, non-specific interstitial pneumonia (NSIP; biopsy-proven in all) and unclassifiable IIPs. IPF and NSIP were diagnosed according to the 2002 American Thoracic Society (ATS)/European Respiratory Society (ERS) IIP statement [9], and HRCT patterns were classified based on the 2011 American Thoracic Society (ATS)/European Respiratory Society (ERS)/Japanese Respiratory Society (JRS)/Latin American Thoracic Association (LATA) IPF guidelines [7]. If HRCT showed a possible or inconsistent usual interstitial pneumonia (UIP) pattern, and a pathologic diagnosis was unavailable, the case was interpreted as unclassifiable IIP according to the 2013 ATS/ERS IIP statement [10]. Patients were excluded on grounds of connective tissue disease or systemic vasculitis, history of exposure to any causative agent of ILD, active pulmonary infection, acute respiratory illness in the preceding 4 weeks, or viable neoplasm. The Kyoto Central Clinic Institutional Review Board approved this study protocol. Written informed consent was not obtained from the participants, because this is a retrospective study using clinical and HRCT data that were accumulated in daily practice.

### Clinical evaluation

Clinical information was collected from medical records. Standardized pulmonary function tests and HRCT were performed on the same day [11]. Equations published for Japanese adults were used to determine the predicted values of each parameter [12].

### HRCT techniques

In each instance, thin-section HRCT was done using a CT scanner with single-detector row (Pronto; Hitachi Medical Corporation, Tokyo, Japan) at 120 kVp, 200 mAs, and 33-cm field of view settings. Axial scans (2 mm thick) were obtained at 10-mm intervals, with a gantry speed of 1.0 s/rotation. No contrast medium was used. In the course of scanning, breath-holding was required after deep inspiration in supine position. Each HRCT image generated a 512 × 512 matrix of numeric data (CT numbers) in Hounsfield units (HU) via standard lung algorithm (filter No. 9). In addition to routine calibration by air and water phantoms, CT numbers were corrected using air density samples from intrathoracic trachea to eliminate effects of X-ray tube aging [13].

### Quantitative CT analysis

**Fig. 1:**
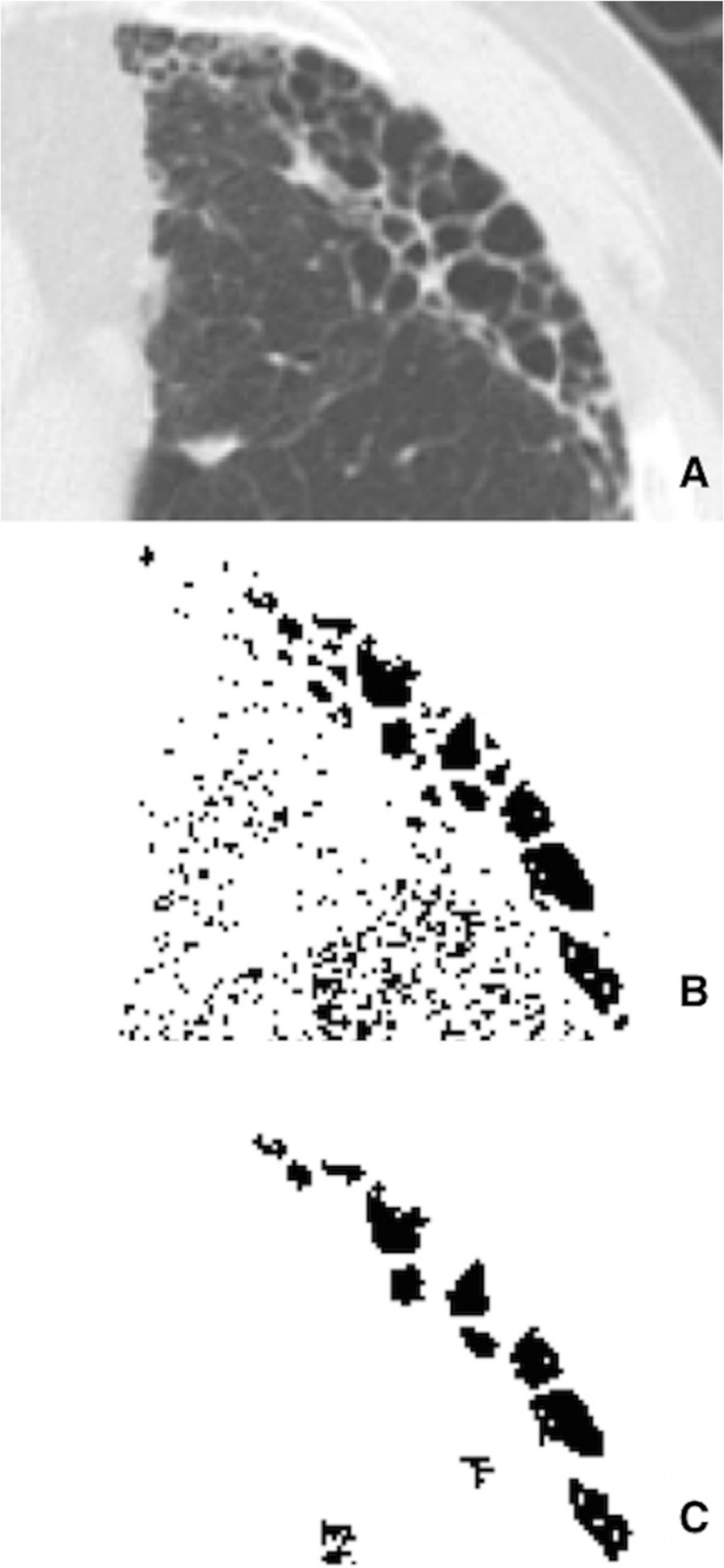
The schema of CT image analysis. **a** Original CT image of the left lung slice. **b** Processed image showing clusters of low attenuation areas (LAA) coloured in black. **c** Processed image showing cystic areas (black areas) defined as LAA clusters with an area of 9π–400π mm^2^

In-house computer software was engaged to analyze all HRCT lung images. Lung fields in each slice were identified by excluding major hilar bronchi and vessels. HAAs and LAAs were defined as areas in lung fields with CT values > −200 HU and < −960 HU, respectively. LAAs were indicative of emphysematous patches, and in general CAs were equated with honeycombing. To extract CAs from LAAs, each LAA cluster was first defined as a continuous LAA entirely bounded by pixels, with CT values > −960 HU. Most emphysematous lesions were small, discrete LAAs or larger LAA clusters, rather than cystic lesions. Cystic lesions only, particularly areas of honeycombing, were defined as LAA clusters with areas of 9π–400π mm^2^ (i.e., circular areas 3–20 mm in diameter) (Fig. 1). The minimum diameter (3 mm) was stipulated by a recent radiologic definition of honeycombing [14], and the maximum diameter (20 mm) was set to exclude continuous emphysematous lesions and bullous changes. HAA% and CA% were calculated as percentages of whole lung field occupied by HAA and CA, respectively. MLD, SD-LD, kurtosis, and skewness likewise were calculated automatically from CT attenuation histograms as follows:

$$ \mathrm{M}\mathrm{L}\mathrm{D}={\sum}_{l = lmin}^{lmax} \ln (l)/N $$

$$ \mathrm{S}\mathrm{D}\hbox{-} \mathrm{L}\mathrm{D}=\sqrt{\sum_{l= lmin}^{lmax}n(l){\left(l-MLD\right)}^2/N} $$

$$ \mathrm{Kurtosis}={\sum}_{l= lmin}^{lmax}\left\{n(l){\left(l-MLD\right)}^4/N{(SD)}^4\right\} - 3 $$

$$ \mathrm{Skewness}={\sum}_{l= lmin}^{lmax}\left\{n(l){\left(l-MLD\right)}^3/N{(SD)}^3\right\} $$

- l = CT value
- n (*l*) = number of pixels in each CT value
- N = number of pixels in all CT values
- SD = SD-LD

MLD and SD-LD represent the average and standard deviation of the HU of each pixel, respectively. Kurtosis describes how sharply peaked a histogram is when compared with the histogram of a normal distribution. Skewness describes the degree of asymmetry of a histogram, and a long right tail indicates positive skewness.

### CT visual scoring

Three independent observers (KT, TH, and TK) blinded to clinical information reviewed HRCT images. Lung fields were divided into upper, middle, and lower zones at level of carina and at right inferior pulmonary venous confluence, respectively. On a scale of 0–5, each of the three zones was rated for extent of GGO (GGO score) and fibrotic opacity (fibrosis score) [15]. Mean scores for each zone and for the whole lung were calculated jointly by the three observers.

### Statistical analyses

Statistical analyses relied on standard software (JMP v9; SAS Institute Inc., Cary, NC, USA). Each statistical variation in quantitative data was expressed as a single determination ± standard deviation, with statistical significance set at *P* < 0.05. Spearman’s rank correlation test was used to examine the relationships between quantitative CT indices, physiologic measures, and CT visual scores. Group comparisons were made using the Mann–Whitney *U* test. Univariate and multivariate survival regression analyses were performed to assess prognostic value of each CT index, applying the Cox proportional hazards model.

## Results

### Characteristics of study patients

Characteristics of study population are summarized in Table 1. Among the 75 patients with fibrotic IIPs, the diagnoses were IPF (*n* = 36, 12 biopsy proven), non-specific interstitial pneumonia (NSIP; *n* = 9, all biopsy proven), and unclassifiable IIP (*n* = 29). On HRCT, definite UIP pattern was observed in 28 (78 %) of IPF group members; and of 29 subjects with unclassifiable IIP, 8 (28 %) displayed possible UIP pattern and 21 (72 %) inconsistent UIP. Mean interval from diagnosis to HRCT evaluation was 58.1 months (range, 5–86 months).

**Table 1:** Characteristics of study population (*n* = 74)

Characteristics	Value	Range
Demographics		
Age, years	65.7 ± 9.2	(30 - 86)
Male	49 (66 %)	-
Smoking (yes)	45 (60 %)	
Interval from diagnosis, months	58.1 ± 48.2	(1 - 263)
Diagnosis		
IPF	36 (49 %)	
NSIP	9 (12 %)	
Other	29 (39 %)	
Pulmonary function tests		
%FVC, %	72.2 ± 20.0	(29.0 - 113.3)
%DLCO, %	54.1 ± 21.8	(10.3 - 122.9)
Quantitative CT indices		
HAA% (>-200 HU), %	1.83 ± 1.15	(0.27 - 7.01)
LAA% (<-960 HU), %	3.91 ± 4.25	(0.14 - 22.5)
CA%, %	0.94 ± 1.85	(0 - 10.54)
MLD, HU	−758.0 ± 53.3	(−860.3 - −635.7)
SD-LD, HU	195.9 ± 22.6	(152.5 - 259.4)
Kurtosis	2.40 ± 1.81	(−0.53 - 7.45)
Skewness	1.61 ± 0.44	(0.66 -2.68)
CT visual scores		
GGO score [0-5]	0.45 ± 0.52	(0 - 2.06)
Fibrosis score [0-5]	1.66 ± 0.68	(0.33 - 3.28)
Fibrosis score ≥2 in any field	48 (65 %)	

Values expressed as mean ± standard deviation or number (%)

Numbers in square brackets represent theoretical score range

*IPF* idiopathic pulmonary fibrosis, *NSIP*, nonspecific interstitial pneumonia; *%FVC* percentage of predicted forced vital capacity, *%DLCO* percentage of predicted diffusion capacity of carbon monoxide, *CT* computed tomography, *HAA%* percentage of lung field occupied by high-attenuation areas, *LAA%* percentage of lung field occupied by low-attenuation areas, *CA%* percentage of lung field occupied by cystic areas, *MLD* mean lung density, *HU* hounsfield units, *SD-LD* standard deviation of lung densities, *GGO* ground-glass opacity

### Comparisons of quantitative CT indices, physiologic measures, and CT visual scores

The relationships of quantitative CT indices with physiologic impairment and semi-quantitative visual assessment of CT images are presented in Table 2. The interobserver correlation coefficients of the GGO and fibrosis scores calculated by the Blant-Altoman method were 0.72–0.83 and 0.77–0.95, respectively. The interobserver Spearman’s rank correlation coefficients (r_s_) of the GGO and fibrosis scores were 0.66–0.77 and 0.77–0.96, respectively. HAA% correlated moderately with physiologic measures (|r_s_| = 0.43-0.59) and weakly with fibrosis score (|r_s_| = 0.25). Densitometric parameters correlated moderately with both physiologic measures (|r_s_| = 0.45-0.59) and fibrosis score (|r_s_| = 0.28-0.33), whereas CA% correlated weakly with fibrosis score (r_s_ = 0.35) and held no relationship with physiologic impairment.

**Table 2:** Spearman’s rank correlation coefficients for determinants of fibrotic IP

	HAA%	CA%	MLD	SD-LD	Kurtosis	Skewness
%FVC	–0.59	–	−0.55	−0.52	0.48	0.49
%DLCO	−0.43	–	−0.56	−0.45	0.57	0.59
GGO	–	−0.40	–	–	–	–
Fibrosis	0.25	0.38	–	0.33	−0.28	−0.28

Missing data (–) indicating correlation was not statistically significant

*CT* computed tomography, *HAA%* percentage of lung field occupied by high-attenuation areas, *CA%* percentage of lung field occupied by cystic areas, *MLD* mean lung density, *HU* hounsfield units, *SD-LD* standard deviation of lung densities, *%FVC* percentage of predicted forced vital capacity, *%DLCO* percentage of predicted diffusion capacity of carbon monoxide, *GGO* visual score of ground-glass opacity, Fibrosis, visual fibrosis score

### Comparison of quantitative CT indices in IPF and non-IPF groups

To determine whether quantitative CT indices reflected differences in clinical diagnosis, IPF (*n* = 36) and non-IPF (*n* = 38, i.e., NSIP and unclassifiable IIPs) group values were compared (Table 3). CA% of the IPF group significantly exceeded that of the non-IPF group (1.50 ± 2.41 % vs. 0.41 ± 0.80 %; *P* < 0.01), but HAA% and densitometric parameters of IPF and non-IPF groups were similar.

**Table 3:** Comparison of subject groups: IPF vs non-IPF

	IPF (*n* = 36)	Non-IPF (*n* = 38)	*P*
Pulmonary function tests			
%FVC, %	71.5 ± 20.7	72.9 ± 19.5	NS
%DLCO, %	56.0 ± 23.9	52.5 ± 19.9	NS
Quantitative CT indices			
HAA% (>-200 HU), %	1.90 ± 1.31	1.76 ± 0.99	NS
LAA% (<-960 HU), %	5.25 ± 5.17	2.64 ± 2.62	<0.01
CA%, %	1.50 ± 2.41	0.41 ± 0.80	<0.01
MLD, HU	−767.4 ± 51.0	−749.1 ± 54.6	NS
SD-LD, HU	197.6 ± 24.6	194.3 ± 20.7	NS
Kurtosis	2.54 ± 1.87	2.21 ± 1.82	NS
Skewness	1.67 ± 0.44	1.56 ± 0.45	NS
CT visual scores			
GGO score [0-5]	0.21 ± 0.25	0.67 ± 0.61	<0.01
Fibrosis score [0-5]	2.10 ± 0.44	1.23 ± 0.59	<0.01
Fibrosis score ≥2 in any field	35 (97 %)	13 (34 %)	<0.01

Values expressed as mean ± standard deviation or number (%)

Numbers in square brackets represent theoretical score range

*IPF* idiopathic pulmonary fibrosis, *NSIP* nonspecific interstitial pneumonia, *%FVC* percentage of predicted forced vital capacity, *%DLCO* percentage of predicted diffusion capacity of carbon monoxide, *CT* computed tomography, *HAA%* percentage of lung field occupied by high-attenuation areas, *LAA%* percentage of lung field occupied by low-attenuation areas, *CA%* percentage of lung field occupied by cystic areas, *MLD* mean lung density, *HU* hounsfield units, *SD-LD* standard deviation of lung densities, *GGO* ground-glass opacity

Quantitative CT indices of subjects with definite UIP pattern (*n* = 35) and those with other patterns (*n* = 39) on HRCT (Table 4) were also compared. Again, CA% was significantly higher in patients with definite UIP pattern (1.48 ± 2.38 % vs. 0.55 ± 1.19 %; *P* < 0.01), whereas HAA% and densitometric parameters did not differ between groups.

**Table 4:** Comparison of subject groups: definite UIP (HRCT) vs other patterns

	Definite UIP (*n* = 35)	Other patterns (*n* = 39)	*P*
Pulmonary function tests			
%FVC, %	71.7 ± 20.6	73.0 ± 19.4	NS
%DLCO, %	54.8 ± 23.4	53.5 ± 20.6	NS
Quantitative CT indices			
HAA% (>-200 HU), %	1.93 ± 1.32	1.74 ± 0.98	NS
LAA% (<-960 HU), %	5.34 ± 5.22	2.63 ± 2.59	<0.01
CA%, %	1.54 ± 2.43	0.40 ± 0.79	<0.01
MLD, HU	−765.8 ± 50.9	−751.0 ± 55.2	NS
SD-LD, HU	198.2 ± 24.8	193.9 ± 20.6	NS
Kurtosis	2.50 ± 1.81	2.25 ± 1.82	NS
Skewness	1.66 ± 0.44	1.58 ± 0.45	NS
CT visual scores			
GGO score [0-5]	0.21 ± 0.25	0.66 ± 0.61	<0.01
Fibrosis score [0-5]	2.13 ± 0.41	1.23 ± 0.50	<0.01
Fibrosis score ≥2 in any field	35 (100 %)	13 (33 %)	<0.01

Values expressed as mean ± standard deviation or number (%)

Numbers in square brackets represent theoretical score range

*IPF* idiopathic pulmonary fibrosis, *NSIP* nonspecific interstitial pneumonia, *%FVC* percentage of predicted forced vital capacity, *%DLCO* percentage of predicted diffusion capacity of carbon monoxide, *CT* computed tomography, *HAA%* percentage of lung field occupied by high-attenuation areas, *LAA%* percentage of lung field occupied by low-attenuation areas, *CA%* percentage of lung field occupied by cystic areas, *MLD* mean lung density, *HU* hounsfield units, *SD-LD* standard deviation of lung densities, *GGO* ground-glass opacity

### Prognostic value of quantitative CT analysis

Median duration of follow-up after HRCT was 38 months (range, 5–86 months), and survivors were followed up for 8–86 months (median, 66 months). Of subjects studied, 31 (41 %) died (chronic respiratory failure, 13; acute exacerbation, 12; pulmonary infections, 4; pneumothorax, 1; sudden death, 1).

Univariate regression analyses indicated that HAA%, MLD, SD-MLD, kurtosis, skewness, fibrosis score, and definite UIP pattern corresponded significantly with poorer prognosis (Table 5), whereas CA% was not a significant predictor of mortality. Given these results, HAA%, kurtosis, fibrosis score, and definite UIP pattern were entered into the Cox proportional hazards model. Kurtosis was the sole densitometric parameter entered, because it correlated strongly with other parameters (|r_s_| = 0.85-0.98), and another study in IPF recognized its superiority in predicting mortality [5]. In multivariate analysis, kurtosis was the only significant determinant of prognosis (HR = 0.67; 95 % CI, 0.44-0.96; *P* = 0.03) (Table 5).

**Table 5:** Univariate and multivariate regression analyses: radiologic indices of survival (*n* = 74)

	Univariate			Multivariate		
	HR	95 % CI	*P*	HR	95 % CI	*P*
Quantitative CT indices						
HAA% (>-200 HU), %	1.47	(1.13 – 1.83)	< 0.01	1.06	(0.86 – 1.06)	NS
LAA% (<-960 HU), %	0.97	(0.86 – 1.06)	NS			
CA%, %	0.98	(0.75 – 1.16)	NS			
MLD, HU	1.01	(1.00 – 1.02)	0.01			
SD-LD, HU	1.03	(1.01 – 1.04)	< 0.01			
Kurtosis	0.67	(0.51 – 0.86)	< 0.01	0.67	(0.44 – 0.96)	0.03
Skewness	0.28	(0.11 – 0.65)	< 0.01			
CT visual scores						
GGO score	0.74	(0.35 – 1.40)	NS			
Fibrosis score	2.00	(1.20 – 3.39)	0.01	1.06	(0.45 – 2.21)	NS
Definite UIP pattern	2.13	(1.05 – 4.47)	0.04	2.36	(0.84 – 7.60)	NS

*HR* hazard ratio, *CI* confidence interval, *CT* computed tomography, *HAA%* percentage of lung field occupied by high-attenuation areas, *CA%* percentage of lung field occupied by cystic areas, *MLD* mean lung density, *HU* hounsfield units, *SD-LD* standard deviation of lung densities, *GGO* ground-glass opacity, *UIP* usual interstitial pneumonia

To assess the independent prognostic value of kurtosis, multivariate regression analyses were performed, coupling kurtosis with a second clinical parameter (Table 6). Kurtosis remained a significant prognostic factor, even after adjusting for age, male gender, and clinical diagnosis of IPF separately (Table 6, models 1–3). Kurtosis was also a stronger predictor of mortality than %FVC (Table 6, model 4), albeit surpassed by %DLCO.

**Table 6:** Multivariate survival regression analyses: kurtosis and other clinical parameters

Model 1			
	HR	95 % CI	*P*
Age	1.01	(0.97 – 1.06)	0.54
Kurtosis	0.67	(0.51 – 0.86)	<0.01
Model 2			
	HR	95 % CI	*P*
Male	2.96	(1.23 – 8.78)	0.01
Kurtosis	0.66	(0.50 – 0.85)	<0.01
Model 3			
	HR	95 % CI	*P*
IPF	2.85	(1.37 – 6.17)	<0.01
Kurtosis	0.62	(0.46 – 0.81)	<0.01
Model 4			
	HR	95%CI	*P*
%FVC	0.98	(0.96 – 1.00)	0.05
Kurtosis	0.75	(0.55 – 0.97)	0.03
Model 5			
	HR	95 % CI	*P*
%DLCO	0.96	(0.93 – 0.99)	0.01
Kurtosis	0.81	(0.57 – 1.07)	0.15

*HR* hazard ratio, *CI* confidence interval, *IPF* idiopathic pulmonary fibrosis, *%FVC* percentage of predicted forced vital capacity, *%DLCO* percentage of predicted diffusion capacity of carbon monoxide

## Discussion

Through this study, we found that HAA% and densitometric parameters were associated with physiologic measures and with CT visual scores in fibrotic IP, whereas CA% helped to distinguish between IPF and non-IPF diagnoses. High HAA% was also associated with survival, but among CT indices, kurtosis was the most significant predictor of mortality. These outcomes validate use of HAA% and CA% as indices with which to quantify parenchymal lesions of fibrotic IP in CT images.

A novel CT index, HAA%, was introduced herein to gauge the extent of parenchymal abnormalities (primarily fibrotic lesions) in fibrotic IP [8]. Restrictive pulmonary function (reduction in %FVC) and impaired gas exchange (diminished %DLCO) are the major physiologic impairments in fibrotic IP; as anticipated, HAA% corresponded with both, similar to conventional densitometric parameters. Correlations between densitometric parameters and physiologic indices have been reported in IPF [1, 2], in asbestosis [1], and in scleroderma [4]. Our data generated from CT histograms have extended these findings to a more heterogeneous group of fibrotic IIPs, adding HAA% to current battery of available parameters. Because HAA% reflects the extent of parenchymal lesions, such as GGO and reticulation [8], it appears that quantifying the degree of fibrotic changes determines the physiologic burden of fibrotic IP. This concept is aligned with a previous study in IPF where semi-quantitative scoring of fibrotic lesions was done [16]. The impact of HAA% and densitometric parameters on survival, shown in univariate analysis, also validates HAA% as a clinically relevant CT determinant of fibrotic IP.

The second new CT index introduced, largely coinciding with extent of honeycombing, was CA%. Unlike HAA% and densitometric parameters, CA% seemed to reflect clinical diagnostic and radiologic pattern differences and thus may be useful for distinguishing IPF and definite UIP pattern from other entities. The significant correlation shown between CA% and fibrosis score (i.e., visually scored honeycombing) suggests that CA% may capture and quantify characteristic lesions of fibrotic IP. On the other hand, we found no association between CA% and %FVC or %DLCO. Although restrictive impairment and extent of honeycombing did not correlate in other studies of IPF [16, 17], significant correlations between honeycombing and impaired gas exchange were consistently identified, using semi-quantitative scoring [16] and a new quantitative CT method, texture analysis [17]. In terms of survival, our outcomes also differed from these studies, which showed that degree of honeycombing significantly predicted mortality in IPF. There was no relationship between CA% and survival in our cohort. These discrepancies raise the possibility that our method of determining CA% differed somewhat from assessing honeycombing visually, although CA% correlated significantly with fibrosis score in our study. Further studies are needed to define the properties of CA% and to refine the measuring of cystic lesions and honeycombing in fibrotic IP.

Among quantitative CT indices, only MLD failed to correlate with fibrosis score. As honeycombing or LAA expands, fibrosis score increases, whereas MLD may remain unchanged or decrease. The reason is that increments in LAA (including honeycombing) offset any fluctuations in HAA. Although MLD is associated with physiologic measures and survival, this dynamic suggests a possible flaw in using MLD as an integrative index of fibrotic and cystic lesions in fibrotic IP. MLD properties differing from those other densitometric parameters were also reported in distinguishing IPF from NSIP [3].

Our analysis of CT parameters identified kurtosis as the strongest predictor of mortality in fibrotic IP. Univariate analysis also underscored that HAA%, fibrosis score, and definite UIP pattern were significant correlates of survival. These findings approximated those of another study of IPF, showing that kurtosis surpassed other densitometric parameters in this regard, although fibrosis score was the only variable of significance in multivariate analysis [5]. Consequently, it appears that quantitative CT indices, including HAA%, enable assessment of disease burden in fibrotic IP and may signal long-term outcomes, making them potential surrogate markers for clinical trials. In addition, kurtosis was independent of other clinical and physiologic parameters in its prognostic capacity, except for %DLCO. Given the difficulty in measuring %DLCO in patients with severe respiratory failure, these CT indices may more readily serve analogous roles in clinical trials and other settings.

The pathological background of these CT indices has been investigated in prior studies. Do et al. reported that kurtosis and skewness were higher in patients with pathological UIP than in those with NSIP [3]. Sumikawa et al. revealed that the histograms of GGA and fine reticulation patterns were similar, while the honeycombing pattern showed less kurtosis and skewness and a higher contrast and variance [18]. On the other hand, they also showed that the histogram of the whole lung was similar between UIP and NSIP, although an analysis of cubic regions of interest (ROIs) demonstrated differences between UIP and NSIP [19]. Those findings suggest that the whole lungs of patients with ILD are combinations of various ILD-characteristic regions. Although the histogram of each region can reflect the differences among the regions, the features of different ROIs offset each other in the histogram analysis of the whole lung, leading to conflicting results of comparisons between different pathological patterns [3, 20]. Indeed, our results showed no significant differences in densitometric parameters between the IPF and non-IPF groups. Given the significant association between densitometric parameters and physiological impairments and long-term outcomes, the densitometric parameters of the whole lung might represent the physiological burdens of disease rather than pathological patterns. The novel CT indices used in this study, %HAA and %CA, are presumed to reflect the fibrotic and honeycombing lesions, respectively. The %HAA was similar between the IPF and non-IPF groups. Although a different definition was used, the percentages of low, intermediate, and high CT density areas did not differ between UIP and NSIP in a previous study [20]. Similar to the densitometric parameters, the %HAA or high-density area might not be an index for morphological characteristics but might instead be an index for the extent and severity of disease. Of note, the %CA was higher in patients with IPF and correlated with the extent of honeycombing by visual scoring in our study. Those results suggest the possibility that the %CA can detect the pathological features of IPF/UIP even in whole-lung analyses.

Quantitative CT analysis of the lung has been performed for COPD, bronchial asthma, and ILDs [6, 13, 21–26]. Histogram analysis of fibrotic ILDs has been conducted for IPF, asbestosis, and scleroderma [1, 2, 4, 5]; and MLD, SD-LD, kurtosis, and skewness were employed as CT indices in those studies. Sumikawa et al. added contrast, variance, and entropy to the repertory of CT indices used to discriminate the different ILD-characteristic abnormalities more precisely [20]. In contrast to those for COPD and bronchial asthma, the standard CT indices remained to be elucidated for ILDs. In addition to the histogram indices, we calculated the %HAA and %CA. As aforementioned, the analysis of whole-lung histograms might not be able to detect the extents of different disease-characteristic lesions sufficiently, because each lesion can offset other lesions in a single histogram. Therefore, we sought to measure the areas of fibrotic lesions and honeycombing directly and automatically. Although such a cut-off approach using CT values has been well established in COPD and emphysema, its utility and limitations in ILDs should be examined in further studies.

Recently, texture analysis has emerged as a novel method for quantifying fibrotic IP by CT [17, 27]. Texture analysis is based on the histogram analysis of ILD- characteristic findings in small ROIs. That method segments the whole lung into small ROIs, classifies each ROI into one CT pattern such as GGA, reticulation, or honeycombing determined through histogram analysis, and calculates the extent of each CT pattern automatically. As a result, the CT data of the whole lung are converted into a combination of the ROI percentages of the histogram-based CT patterns. Texture analysis aims to overcome the limitations of whole-lung histogram analysis by dividing the whole lung into small ROIs, thus avoiding the summation of the whole lung CT data into a single histogram. The expanse of honeycombed areas and serial changes in abnormalities (reticular and total interstitial) in texture analysis reportedly are significant predictors of mortality in IPF [17, 27]. By comparison, percentages of reticular and honeycombed areas in whole lung fields of texture analysis exceeded corresponding %HAA and %CA values in our study, although the study groups were not comparable [17, 27]. These texture analysis parameters also correlated more strongly with visual scores than did %HAA and %CA [17, 27]. Thus, ILD-specific lesions may be better defined via texture analysis than through our pixel-counting and global histogram approach.

On the other hand, texture analysis is a computer-aided method, relying on recognition of radiologic features by a consensus of experts in each study group; indeed, software applications of the various studies were not uniform [17, 27]. Even among experts, inter-observer agreement on ILD-specific abnormalities, such as honeycombing, is less than satisfactory [28]. Thus, a gold standard of analytics has yet to be established, and the broader utility of texture analysis should be examined not only in IPF but also in other IIPs or fibrotic IP.

Another issue in quantitative CT analysis is the selection of ROIs. Sumikawa et al. reported that three-dimensional (3D) histogram analysis using cubic ROIs is superior to two-dimensional histogram analysis with square ROIs for assessing various CT patterns of ILDs [18]. They applied a similar method to quantify pulmonary adenocarcinoma with GGA and demonstrated the utility of 3D histogram analysis in small lung cancer [29]. In addition, a 3D approach was used in a recent texture analysis [27]. We used CT scans with a 2 mm thickness obtained at 10 mm intervals and therefore could not apply a 3D analysis. Those differences in CT scanning conditions might have influenced our results.

There are acknowledged limitations to this study. First, the subjects did not receive uniform treatment such as corticosteroids, immunosuppressive agents, or pirfenidone, because evidence-based guidelines for IPF were just published recently [7]. Given the relatively poor outcomes of the patients with IPF who received the combination therapy of prednisone and azathioprine in the PANTHER-IPF study [30], different therapeutic strategies might have affected the long-term outcomes of patients with IPF. In addition, our cohort included several patients with non-IPF IIPs, whose responses to treatment could be more variable. Hence, we could not address the impact of therapeutic regimens on survival. Furthermore, the ramifications of dyspnea, overall health status, exercise capacity, and comorbidities could not be assessed, due to the retrospective design. These factors may well have bearing on prognosis of IPF and fibrotic IIPs [31–37]. Additionally, our cohort included several patients with unclassifiable IIP because of a lack of pathological diagnoses. Those patients can be potentially diagnosed with IPF or NSIP, and such diagnoses might influence the results of comparisons between IPF and non-IPF and multivariate survival analyses. Finally, we did not determine longitudinal changes in CT indices at this time. Best et al. have already demonstrated that serial changes in densitometric parameters correlate with changes in physiologic measures [5]. Given the significant impact of declining %FVC on survival of patients with IPF [7, 36, 38, 39], the prognostic implications of changes in quantitative CT indices, particularly HAA%, should be examined in future studies.

## Conclusion

Despite these limitations, the differing properties of these novel quantitative CT indices, HAA% and CA%, were evident. HAA% largely reflected physiologic impairments in fibrotic IP, whereas CA% corresponded with diagnosis and HRCT pattern. Future studies applying quantitative CT analysis for fibrotic IP should incorporate these CT indices to assess disease characteristics and severity more comprehensively.

## Abbreviations

%DLCO: Percentage of predicted diffusion capacity of carbon monoxide
%FVC: Percentage of predicted forced vital capacity
CA%: Percentages of cystic areas
CAs: Cystic areas
CI: Confidence interval
CT: Computed tomography
GGO: Ground-glass opacity
HAA%: Percentages of high-attenuation areas
HAAs: High-attenuation areas
HR: Hazard ratio
HRCT: High-resolution computed tomography
HU: Hounsfield units
IIP: Idiopathic interstitial pneumonia
ILDs: Interstitial lung diseases
IP: Interstitial pneumonia
IPF: Idiopathic pulmonary fibrosis
LAA%: Percentage of lung field occupied by low-attenuation areas
LAAs: Low-attenuation areas
MLD: Mean lung density
NSIP: Non-specific interstitial pneumonia
r_s_: Spearman’s rank correlation coefficient
SD-LD: Standard deviation of lung density
UIP: Usual interstitial pneumonia
